# [Corrigendum] MicroRNA-122 acts as tumor suppressor by targeting TRIM29 and blocking the activity of PI3K/AKT signaling in nasopharyngeal carcinoma *in vitro*

**DOI:** 10.3892/mmr.2026.13879

**Published:** 2026-04-15

**Authors:** Yan Yang, Qing Li, Lili Guo

Mol Med Rep 17: 8244–8252, 2018; DOI: 10.3892/mmr.2018.8894

Subsequently to the publication of the above article, an interested reader drew to the authors' attention that, concerning the cell migration and invasion assay experiments in Fig. 2C and D on p. 8248, a number of data panels contained overlapping sections, such that data which were shown to represent different experimental conditions had been derived from a smaller number of orginal sources.

Given the time that has elapsed since this paper was published, the authors no longer had access to their original data, but were willing to repeat these experiments, and the revised version of Fig. 2 is shown on the next page. The results obtained were broadly similar to those obtained originally, and therefore the replacement of the original data in this figure with the new data does not affect the reported conclusions of this study. All the authors agree with the publication of this Corrigendum, and are grateful to the Editor of *Molecular Medicine Report*s for allowing this to be published. They also regret the errors that existed in the original figure, and apologize to the readership for any confusion that may have been caused.

## Figures and Tables

**Figure 2.**
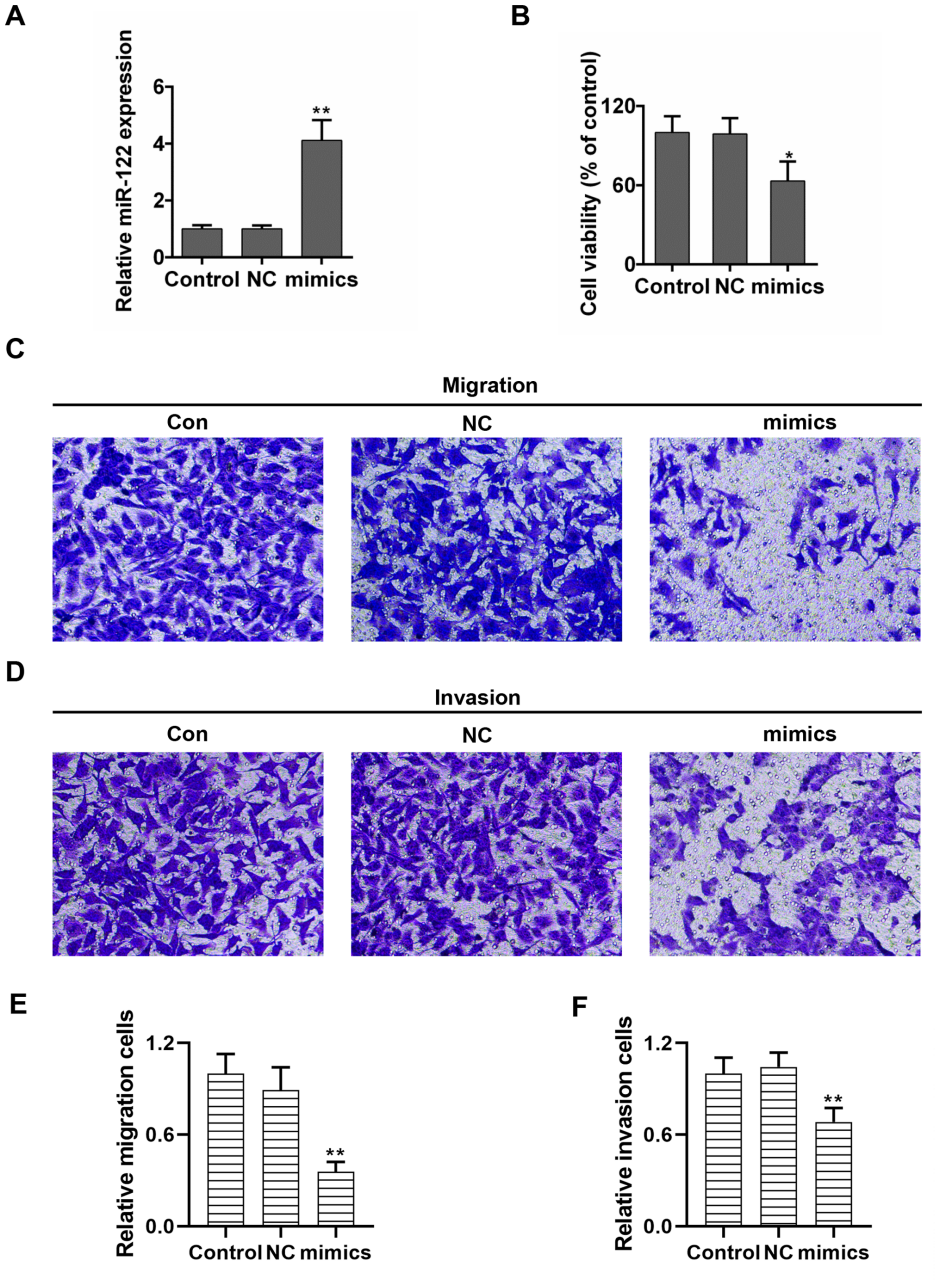
miR-122 suppresses cell proliferation, migration and invasion in NPC cells. (A) The significantly elevated expression of miR-122 in NPC cells transfected with miR-122 mimics. (B) Relative cell survival, demonstrated using Cell Counting kit-8 methods. Data are presented as the mean ± standard deviation, n=3. *P<0.05 and **P<0.01 vs. control. Representative images of the (C) migratory and (D) invasive abilities of NPC cells transfected with miR-122 are presented (magnification, ×100). (E) miR-122 inhibited the migratory ability of NPC cells. (F) miR-122 inhibited the invasive ability of NPC cells. Data are presented as the mean ± standard deviation, n=4. **P<0.01 vs. control. NPC, nasopharyngeal carcinoma; miR, microRNA; NC, negative control; Con, control.

